# Phenology and related traits for wheat adaptation

**DOI:** 10.1038/s41437-020-0320-1

**Published:** 2020-05-26

**Authors:** Jessica Hyles, Maxwell T. Bloomfield, James R. Hunt, Richard M. Trethowan, Ben Trevaskis

**Affiliations:** 1 The Plant Breeding Institute, University of Sydney, 107 Cobbity Road, Cobbity, NSW 2750 Australia; 2 CSIRO Agriculture and Food, GPO Box 1700, Canberra, ACT 2601 Australia; 3 Department of Animal, Plant and Soil Sciences, La Trobe University, 5 Ring Road, Bundoora, VIC 3083 Australia

**Keywords:** Shoot apical meristem, Agricultural genetics, Plant breeding

## Abstract

Wheat is a major food crop, with around 765 million tonnes produced globally. The largest wheat producers include the European Union, China, India, Russia, United States, Canada, Pakistan, Australia, Ukraine and Argentina. Cultivation of wheat across such diverse global environments with variation in climate, biotic and abiotic stresses, requires cultivars adapted to a range of growing conditions. One intrinsic way that wheat achieves adaptation is through variation in phenology (seasonal timing of the lifecycle) and related traits (e.g., those affecting plant architecture). It is important to understand the genes that underlie this variation, and how they interact with each other, other traits and the growing environment. This review summarises the current understanding of phenology and developmental traits that adapt wheat to different environments. Examples are provided to illustrate how different combinations of alleles can facilitate breeding of wheat varieties with optimal crop performance for different growing regions or farming systems.

## Introduction

Genes for phenology and plant development, their interactions with each other and the environment largely determine if a wheat (*Triticum aestivum* L.) crop is successful. For instance, in order to reach maximum seed size and number (potential yield), wheat must establish, develop biomass and flower at a time that coincides with optimal seasonal conditions (Trethowan 2014). Flowering in winter risks frost damage to reproductive structures, and suboptimal radiation levels can reduce yield (Dreccer et al. 2018). Alternatively, if crops flower too late in warm and dry environments, heat damage and water limitation can reduce yield (Flohr et al. 2017). Other aspects of plant biology beyond development are important for adaptation, including winter hardiness and plant architecture, and these must also be co-ordinated with seasonal development.

Understanding the genetic basis for variation in phenology and other adaptive traits can inform crop breeding strategies and contribute to prediction of yield risks, such as drought, frost or heat, and thereby improve crop management. This review focusses on the molecular genetics of wheat adaptation, and how this knowledge can facilitate breeding wheat adapted to diverse growing environments or different farming systems.

## Defining and measuring wheat development

**Fig. 1:**
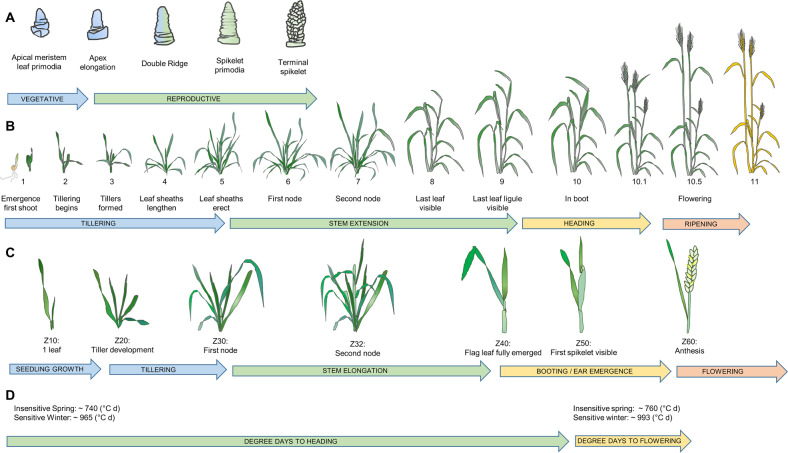
Development stages in wheat. **a** Apex morphology, vegetative to reproductive (Moncur 1981). **b** Feekes Scale, stage 1–11 (Feekes 1941; Large 1954). **c** Zadoks Decimal Scale, score 0–100 (Zadoks et al. 1974). **d** Example of cumulative degree-days from emergence to heading and emergence to flowering for near-isogenic lines (NIL)s with differing vernalisation or photoperiod requirements grown in inductive conditions (Bloomfield et al. 2018 and pers. comm.).

Development is the progression of the plant lifecycle, independent of growth that is due to accumulation of biomass. Development comprises distinct phases outlined in Fig. 1. Feekes developed a scale (stages 1–11) classifying the wheat lifecycle from tillering, stem elongation, heading and flowering, through to ripening (Fig. 1b.) Another developmental scale developed by Haun (1973) quantifies progressive leaf emergence on the main stem of wheat, which can then be used to determine leaf emergence rate, otherwise known as phyllochron. In addition, a comprehensive scale describing the wheat lifecycle from germination through to ripening in a two-digit computer-compatible decimal format was developed (Zadoks et al. 1974). The “Zadoks scale” comprises 100 stages describing development of the wheat plant (Fig. 1c).

The wheat seed usually contains four leaf primordia, and more develop on the vegetative meristem during seedling growth (Z10–Z19). Leaf primordia appear as ridges on the apex, before elongation and differentiation into leaves. The position of emerging leaves is predictable with each new leaf developing on the opposing side of the apex to its predecessor. When around three non-embryonic leaves have developed, tiller buds located in the axils of leaves differentiate to produce tillers (branches) sequentially: tiller 1 from the axil of leaf 1, tiller 2 from leaf 2 and so on. Exceptions to this ordered leaf and tiller development have been described and may be environmentally dependent (Percival 1921). Each tiller has potential to produce secondary tillers. The overall extent of branching, tiller survival and fertility are affected by temperature, light, nutrient status and row spacing. Genetic control of tillering has also been identified (Hyles et al. 2017; Zhao et al. 2019). The primary stem continues to produce tillers, until the plant transitions to the stem elongation (reproductive) phase.

A pivotal point in the wheat lifecycle is transition of the shoot apex from vegetative to reproductive development (Fig. 1a, Waddington et al. 1983). At this stage, production of new leaf primordia ceases, and spikelet formation begins. This represents a commitment to flowering and determines the final leaf number (Wang et al. 1995). The shoot apex elongates, followed by formation of two ridges on the sides of the shoot apex, where previously only single ridges were formed. These can be visualised microscopically; when the plant has reached double-ridge stage, vegetative-to-reproductive transition is complete (Slafer et al. 2015). The lower ridge is a leaf primordium that will later abort, while the upper ridge is the spikelet primordium that will differentiate to form all the floret organs: glume, lemma, palea and stamens of the floret (Moncur 1981). Subsequently, the terminal spikelet forms, and thereafter no further spikelets are formed on the primary axis. The duration of development from double ridge to the terminal spikelet stage is the primary determinant of maximum spikelet number (Rawson 1970).

Simultaneous to early stages of reproductive shoot apex development, stem elongation proceeds. Nodes formed during vegetative development thicken and become a point of rapid growth and extension to form internodes, with each successive internode longer than its predecessor (Evans 1975). This provides a means for the developing spike to travel upwards through the stem from Z30 onwards. As the stem elongates, spikelet differentiation and floret development also occur. Wheat adjusts its growth in response to environmental stress so that the last-formed spikelets at the base and tip are the first to abort in poor-growing conditions. Usually up to 12 floret primordia are formed in each spikelet; however, only 3–5 survive and set seed, thought to be a function of the competition for resources between spikes and stems during the elongation phase (Kirby 1988).

Synchrony of each developmental phase with optimal seasonal conditions is necessary to optimise production of biomass and yield. For instance, grain number and thus yield in wheat is largely determined by growth rates during the critical period that extends from emergence of the penultimate leaf until early grain filling (Dreccer et al. 2018). Agronomically, it is thus vital to align this sensitive stage to the likely occurrence of seasonal conditions (temperature, radiation and water availability) most conducive to wheat growth. The timing of developmental phases also influences abiotic stress tolerance such as winter hardiness. Seasonal conditions and regional factors, including available moisture, temperature, latitude and day length, all influence the duration of developmental phases (Slafer and Rawson 1994; Angus and Moncur 1977; Amir and Sinclair 1991; Trethowan et al. 2006). This dependence of crop phasic development upon the growing environment represents a strong genotype by environment interaction, and acts to synchronise the lifecycle with external conditions.

Early studies demonstrated that the switch from vegetative to reproductive development is promoted by prolonged cold temperatures of winter (vernalisation) (Chouard 1960). The duration of cold is important, for instance, a plant that responds by flowering after a “cold snap” in autumn would not survive in climates with long, cold winters. From a developmental perspective, vernalisation influences the duration of the vegetative phase, and is a large determinant of the final leaf number. Vernalisation requirement is typically combined with day-length-responsive flowering, such that plants that have vernalised over winter will flower rapidly as days subsequently lengthen in spring (Chouard 1960). This led to the “long-day” and “winter-type”, classification of wheat. That is, the naturally occurring ancestral plant type (wild type) requires vernalisation followed by increasing photoperiod in order to flower. In regions with cold winters, autumn sowing of these types allows flowering to coincide with favourable temperatures and radiation in early summer for optimum yield.

## Interaction of plant development and the environment

Alternative life-cycle strategies facilitate adaptation to different environments (Evans et al. 1975). Unlike winter types, spring wheats require little-to-no environmental inducement for flowering (Chouard 1960). These types typically flower rapidly without vernalisation, with rapid progression to the double-ridge stage, and reduced final leaf number relative to winter wheat in similar growing conditions. Spring wheat can also have varying levels of sensitivity to day length. Day-length-insensitive spring cultivars can progress to the terminal spikelet stage and flower rapidly even in short days. Taken together, the absence of vernalisation or day-length requirements allows some spring wheats to be sown in environments with milder winters and at different times of the year (see “Quantitative traits in the farming system”, Fig. 3). From a study of wild emmer wheat *Triticum dicoccoides*, it is thought that spring types evolved from wild-type winter habit in the progenitor of cultivated hexaploid wheat (Kato et al. 1997).

Since wheat can be grown across diverse environments and at different times of the year, it is useful to calibrate development versus temperature and day length using accumulated thermal time or degree-days (DD), or photo-degree days (PDD). This allows comparison of developmental rates across different conditions, where the rate of development per se differs. For DD (Eq. 1), calculations are based on accumulated temperature above a base, and may also consider an upper limit so that only temperatures conducive to plant development are considered. DD can be determined by summing daily average temperatures as the equation below, or considers more frequent measures of temperature or estimates thereof, for example, using sine curve or triangular equations (McMaster and Wilhelm 1997; Zalom et al. 1983; Snyder 1985).

1 $$DD\left( {ave} \right) = \left[ {\frac{{Tmax - Tmin}}{2}} \right] - Tbase.$$

Equation 1. Estimation of thermal time

*DD*(*ave*) = Degree-days, average calculation (°C d)

*Tmax* = Maximum daily temperature (°C)

*Tmin* = Minimum daily temperature (°C)

*Tbase* = Base temperature, typically 0 °C or 5 °C, dependent on growth stage

In a study by Bloomfield et al. (2018), development of near-isogenic lines (NILs) was recorded (in DD) under inductive growth conditions; the approximate cumulative DDs to heading and flowering relative to other scales of development are shown in Fig. 1d (Bloomfield, pers. comm.). Comparison of slow-developing wheats (photoperiod-sensitive winter types) versus fast-developing wheats (photoperiod-insensitive spring types) illustrates the variation in response to temperature between these different classes.

To determine PDD, cumulative time from sunrise to civil twilight (day length) can be incorporated through the following equation (Wilsie 1962):

2 $$PDD = DD\,\times\,t.$$

Equation 2. Estimation of photo-thermal time

*PDD* = Photo-degree days (°C d h)

*DD* = Degree-days (°C d)

*t* = day-length (h)

A similar approach can be applied to calibrate temperature accumulation during vernalisation. Vernal days, the cumulative time in days until vernalisation saturation is reached (i.e., double-ridge stage reached), are determined by summing days from germination to development of the final leaf (Robertson et al. 1996). Porter and Gawith (1999) suggest that vernalisation occurs most rapidly at 4.9 °C, and requires temperatures between −1.3 and 15.7 °C.

### Molecular pathways of wheat development

**Fig. 2:**
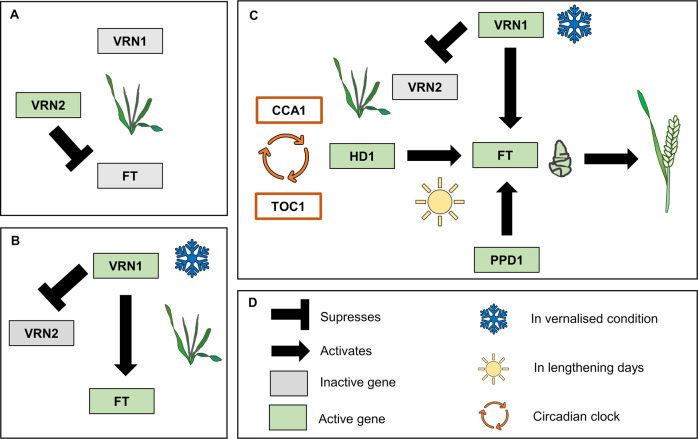
Major genes of the flowering pathway. **a** Gene activity prior to vernalisation (winter wheat). **b** Gene activity in response to vernalisation. **c** Gene activity in response to vernalisation and long days, associated with transition to flowering. **d** Black arrows depict gene action, colour (grey vs. green) illustrates gene expression state (inactive vs. active) and circular arrows represent the feedback loop.

Since the phenology of wheat determines adaptation to different environments, an understanding of the genes underlying developmental variation is paramount. The major genes affecting wheat phenology (see Fig. 2) are those related to vernalisation requirement, photoperiod sensitivity and earliness per se, which is the duration of development until flowering, in conditions where vernalisation and photoperiod requirements are met.

## Vernalisation pathway

The key component of vernalisation requirement of wheat is the *VERNALIZATION1 (VRN1)* locus, with a copy on the long arm of chromosome 5, in each of the A, B and D sub-genomes. *VRN1* encodes an MIKC-type MADS box (MINICHROMOSOME MAINTENANCE1/AGAMOUS/DEFICIENS/SERUM RESPONSE FACTOR), with a conserved 60 amino-acid MADS box DNA- binding domain and three additional domains I (intervening), K (keratin-like) and a C-terminal domain. *VRN1* is most like the *APETALA1/FRUITFULL* class *(AP1/FUL)* of MADS box genes of *Arabidopsis thaliana*. These genes play important roles in floral development in *Arabidopsis,* and can trigger early flowering when expressed at high levels (Mandel and Yanofsky 1995). Unlike the *AP1/FUL* genes of *Arabidopsis*, transcription of *VRN1* increases with exposure to prolonged cold (Danyluk et al. 2003; Trevaskis et al. 2003; Yan et al. 2003). It seems that *VRN1* evolved from recruitment of the floral-promoting potential of *AP1/FUL* genes to provide a low-temperature-induced flowering switch. This role for *AP1/FUL-*like genes is seemingly unique to the temperate grasses. *VRN1* is expressed in both leaves and shoot apices of vernalised plants; accumulation of *VRN1* transcripts in the shoot apex is associated with the switch to reproductive development, while transcription of *VRN1* in leaves facilitates the long-day flowering response after winter (Fig. 2).

The precise mechanism that mediates low-temperature induction of *VRN1* is not known, but histone modifications appear to play a role. Epigenetic modification of chromatin by histone modification or methylation of DNA has been well studied and linked to heritable changes in gene expression and phenotypic variance (see Banta and Richards (2018) for review). Histone modifications mediate downregulation of the *Arabidopsis* flowering repressor *FLOWERING LOCUS C* during vernalisation (Finnegan et al. 2005). In cereals, before vernalisation, histones at the promoter and the first intron of the *VRN1* locus have modifications associated with gene repression (histone 3 lysine 27 trimethylation, H3K27me3), and during vernalisation, there is a shift towards modifications typical of active genes (histone 3 lysine 4 trimethylation, H3K4me3) (Oliver et al. 2009). These histone modifications potentially maintain repression of *VRN1* before winter and conversely, sustain activity of *VRN1* after prolonged cold. This could provide a “memory” of vernalisation, such that chromatin at the *VRN1* locus remains in an active state after winter even when temperatures rise, allowing flowering to proceed when days lengthen in spring (Oliver et al. 2009). Presumably, the chromatin state is restored during meiosis, as the vernalisation requirement “resets” in progeny. During seed development, cold conditions while ripening can vernalise the progeny seed, and this memory of vernalisation is retained post seed development, drying and harvest (Gregory and Purvis 1936; Atayde 2019). The implications of this need to be considered during seed increases and cropping situations.

Mutations in the promoter and deletions in the large first intron of *VRN1* are both associated with elevated expression of the gene in the absence of cold and accelerated flowering without vernalisation (Kippes et al. 2018). These mutations are found in the *VRN1* gene from each of the A, B and D genomes, and give rise to dominant alleles for reduced vernalisation requirement, with the A-genome version conferring the greatest effect (no requirement for cold temperature to flower) relative to the B- and D-genome alleles (reduced vernalisation requirement, semi-spring types) (Trevaskis et al. 2003). The difference between the sub-genomes is potentially due to the nature of the mutations found in each allele (i.e., promoter insertion plus gene duplication on A genome, intron deletions of differing size on B and D genomes).

The first intron of *VRN1* contains a binding site for the *T. aestivum* glycine-rich RNA-binding protein 2 (TaGRP2), which blocks expression of *VRN1* until it is released by cold. During sustained low temperatures, TaGRP2 interacts with a jacalin lectin carbohydrate-binding protein TaVER2 (vernalisation-related 2) via O-GlcNAc (O-linked β*-N*-acetyl glucosamine) (Xiao et al. 2014). This in turn leads to accumulation of *VRN1* transcripts and ultimately, flowering. Some wheats have sequence variation (single-nucleotide polymorphisms, SNPs) in the TaGRP2-binding site within the first intron of the A-genome copy of *VRN1*. These reduce the binding of TaGRP2, and are associated with a moderate reduction of vernalisation requirement (Kippes et al. 2018; Xu et al. 2019). Loss of TaGRP2-binding sites might partially explain why deletions in the first intron are associated with increased *VRN1* expression.

*VERNALIZATION4 (VRN4)*, which also reduces vernalisation requirement, is located on chromosome 5DS and arose from translocation of the region from chromosome 5A that contains the *VRN1* gene (Kippes et al. 2014). *VRN4* is associated with increased *VRN1* transcript levels from the extra gene copy at the *VRN4* locus, and thus reduced vernalisation requirement. The copy of *VRN1* at the *VRN4* locus contains the intron SNPs described earlier (those which disrupt TaGRP2 binding in *VRN1*), which potentially explains why *VRN1* transcription is elevated in wheats that carry *VRN4*. The origin of *VRN4* in Australian cultivars has been traced to cv. Gabo (Kippes et al. 2015), an important cultivar introducing spring-growth habit and adaptation to the Australian climate.

Other MADS box genes also play roles in regulation of wheat flowering. Two other *AP1/FUL-*like genes *TaFUL2* and *TaFUL3* are paralogues of *VRN1* that regulate spike development and also influence flowering time, though to a lesser extent than *VRN1* (Li et al. 2019). Another MADS box gene, *ODDSOC2* *(OS2)* (also known as *TaAGL33* and *TaAGL22* in wheat) is a repressor of flowering downregulated by vernalisation (Greenup et al. 2010, 2011). A *Short vegetative phase-like* gene, *Vegetative to reproductive transition 2* *(VRT2)*, located on the short arm of group 7 chromosomes, was suggested to be a repressor of floral development downregulated by cold (Kane et al. 2005), but subsequent studies found that transcription of this gene increases at low temperatures, and that *VRT2* more likely activates flowering in cooperation with *VRN1* (Trevaskis et al. 2007; Xie et al. 2019). It remains unclear whether any of these MADS box genes underlie variation in phenology or any other developmental traits.

The gene that triggers long-day-induced flowering of wheat is the functional equivalent of *Arabidopsis FLOWERING LOCUS T (FT)*, referred to here as *TaFT1* for *FT-like 1* (Turner et al. 2005). FT is proposed to be “florigen”, a plant hormone capable of triggering flowering in inductive day-length conditions (Zeevaart 2006). In wheat, *TaFT1* is transcribed in long days, but only when *VRN1* is active (i.e., in vernalised plants or those containing spring *VRN1* alleles, see Fig. 2), consistent with vernalisation being a prerequisite for long-day-induced flowering in winter wheat (Yan et al. 2006). At the shoot apex, the TaFT protein interacts with a bZIP transcription factor (encoded by *FLOWERING LOCUS D-LIKE 2, TaFDL2*). The resulting TaFT protein complex directly binds to the promoter of *VRN1* at sites with an ACGT core motif, and in some genotypes and environments, this promotes further expression of *VRN1* (Distelfeld et al. 2009).

The *VRN3* gene, which can reduce the vernalisation requirement of wheat, has been mapped to *TaFT1* (Yan et al. 2006). Insertions within the promoter of *TaFT1/VRN3* give rise to dominant alleles associated with elevated *TaFT1* expression and rapid flowering, irrespective of vernalisation or day length. Conversely, deletion of the B-genome copy of *TaFT1* delays flowering, extending the spike development phase and increasing spikelet numbers under long-day conditions (Finnegan et al. 2018). Other genes from the *FT* family have been identified in cereals. Shaw et al. (2019) found that *TaFT2* had a mild effect on time to flowering and a more profound effect on spikelet number, while the *FT3* of barley (*Hordeum vulgare*) was associated with accelerated flowering in short-day conditions (Halliwell et al. 2016).

*VRN2* is a repressor of flowering that plays a key role in blocking the long-day flowering response before winter (Fig. 2) (Yan et al. 2004; Trevaskis et al. 2006). The *VRN2* locus contains two closely related “zinc-finger CCT” genes (*ZCCT1* and *ZCCT2*), so-called due to the presence of a zinc finger at the N terminus and a conserved CCT domain first identified in the predicted protein sequences of *CONSTANS*, *CONSTANS-like* and *TIMING OF CAB EXPRESSION-1* genes (Yan et al. 2004; Kippes et al. 2015; Li and Xu 2017). *VRN2* is expressed in long days where it represses flowering by suppressing transcription of *TaFT1* (Trevaskis et al. 2006; Hemming et al. 2008). This repression likely occurs via protein interactions with *NUCLEAR FACTOR* -*Y* *(NF-Y)* genes (Li et al. 2011). NF-Y proteins interact with proteins containing CCT domains and bind to the CCAAT box of promoters to elicit expression responses (Stephenson et al. 2007). Following winter, in vernalised plants, the VRN1 protein is produced and binds to the promoter of *VRN2* and *TaFT1*. This downregulates *VRN2* and so allows the photoperiod pathway to activate *TaFT1* in long days, to promote flowering (Trevaskis et al. 2006; Deng et al. 2015). *VRN1* binding to the promoter of *TaFT1* potentially plays a more direct role in activating the long-day flowering response.

The wild-type “winter” phenotype (requirement for vernalisation) requires a functional copy of *VRN2*. Recessive *VRN2* loss-of-function alleles give rise to spring growth habit in diploid wheats and barley, but in a day-length-dependent matter, where flowering is accelerated in long days. Loss-of-function alleles of *VRN2* in hexaploid wheat are unlikely to account for natural variation in phenology due to genome redundancy masking allele effects; however, triple loss-of-function genotypes of hexaploid bread wheat have been generated by inducing and stacking loss-of-function mutants of all three copies of the *VRN2* gene (Yan et al. 2004; Distelfeld et al. 2009). This suggests that there may be potential for the generation of new allelic diversity at *VRN2* to broaden the adaptation of wheat.

## Photoperiod sensitivity

Sensitivity to day length is largely determined by alleles of the *PHOTOPERIOD1 (PPD1)* gene, with homoeologous copies on the A, B and D genomes of hexaploid bread wheat (chromosomes 2A, 2B and 2D) (Welsh et al. 1973; Law et al. 1978). *PPD1* belongs to a pseudoresponse regulator (PRR) family and is also known as *PRR37*. As for *VRN2*, the PRR family of proteins feature the CCT motif (Mizuno and Nakamichi 2005). Wild-type alleles of *PPD1* have a rhythmic diurnal pattern of gene expression (peak in the middle of the day), and are associated with day-length-sensitivity, where lengthening/longer days accelerate flowering (Diaz et al. 2012, Shaw et al. 2012). Allelic diversity in *PPD1* arises through deletions or a transposon insertion in the promoter, and through copy-number variation (CNV). Diaz et al. (2012) showed that alleles of *PPD-B1* (along with *VRN-A1*) were associated with increased copy number of both genes, and resulted in earlier flowering (*PPD-B1a*) or increased vernalisation requirement (*VRN-A1w*). These results, along with a separate study in durum (Wu̎rschum et al. 2017), suggest that copy-number variation is important for the adaptation of wheat. Non-wild-type alleles of *PPD1* alter the expression of the gene, leading to elevated transcription throughout the day, and accelerated flowering through elevated *TaFT1* expression. This can substitute for long days and reduce day-length sensitivity.

Alleles that confer a strong insensitivity to day length (e.g., an allele of the D-genome copy of *PPD1, PPD-D1a*, with a deletion in the promoter region) are associated with rapid flowering in all day-length conditions (Diaz et al. 2012; Wilhelm et al. 2009). Other studies (Bentley et al. 2011, 2013) describe the importance of the *PPD-A1* and *PPD-B1* loci. It is likely that these differences are driven by allele-specific effects (e.g., the nature of discrete mutations in the *PPD1* gene) rather than simply due to a particular genome. It is worth noting that long days will accelerate flowering to some extent, even in wheats with the alleles of *PPD1* that confer strong day-length insensitivity, suggesting that additional genes or pathways can contribute to the long-day flowering response of wheat (Bloomfield et al. 2018). Similarly, flowering of “day-length insensitive” wheats can be further accelerated by elevated ambient temperatures (Hemming et al. 2012).

Alleles of *PPD1* that are associated with reduced day-length sensitivity are also associated with an increased rate of spikelet development and decreased spike fertility (Prieto et al. 2018). A recent study attributed a shorter duration of pre-anthesis stem elongation and decreased number of fertile florets to *PPD-D1a*, highlighting the scope for increased yield potential by selection for photoperiod-sensitive alleles (Perez-Gianmarco et al. 2019). Conversely, it may be beneficial to select for insensitivity in some environments as *PPD-D1a* has been shown to increase the duration of flowering (across all tillers of a plant, not within a single spike) (Jones et al. 2017). This ability of the plant to spread flowering of tillers could be beneficial to minimise the impact of a brief stress event, such as frost or heat, because not all spikes on the plant will be at a stage most sensitive to damage (Lukac et al. 2012).

Genes at other loci are also part of the photoperiodic flowering pathway. Zhang et al. (2016) reported a *TaPPD1* paralogue, *TaPRR73*, located on chromosome 4A, was highly expressed in early-flowering wheats and contributed to plant height. A locus on chromosome 7B, *TaPPD-B2*, was associated with early flowering in long days, and linked to high protein content of grain (Khlestkina et al. 2009).

## Circadian clock

The importance of *PPD1*, a *PRR* gene, in determining photoperiod sensitivity of wheat, highlights the fundamental role of the circadian clock in coordination of the day-length response (Mizuno and Nakamichi 2005). The circadian clock is the intrinsic mechanism used by plants to synchronise internal biological processes with the daily fluctuating environment that is cycles of light and temperature between day and night (Ford et al. 2016). Aside from a key role in day-length perception, the circadian clock also regulates other important biological processes, such as photosynthesis, metabolism and the response to biotic and abiotic stress, to maintain synchrony between internal processes and daily changes in the external growing environment.

The plant circadian clock has been studied intensively in *Arabidopsis*, with much less research undertaken in wheat. The fundamental components appear conserved in cereals, so *Arabidopsis* remains an exemplar model. The clock consists of negative feedback loops that give rise to rhythmic waves of gene expression through the day–night cycle (see Hsu and Harmer 2014 for review). *Circadian clock-associated 1 (CCA1)* and *late-elongated hypocotyl* *(LHY)* are MYB transcription factor genes with peak transcript levels occurring at dawn. Then there are a series of *PRR* genes that are expressed sequentially from morning to evening (*PRR9, PRR7, PRR5, PRR3* and *TIMING OF CAB EXPRESSION 1* *(TOC1)*). In the morning, *CCA1* and *LHY* repress transcription of *TOC1* (also known as *PRR1*). *TOC1* expression peaks in the evening, and this in turn represses *CCA1* and *LHY*, creating a feedback loop. Other components of the circadian clock include *ARRHYTHMO/PHYTOCLOCK (LUX/PCL), EARLY FLOWERING 3 (ELF3), EARLY FLOWERING 4 (ELF4)* and *GIGANTEA (GI)* (see Bendix et al. (2015) for review).

The circadian clock of *Arabidopsis* plays a key role in the photoperiod pathway by regulating diurnal expression of *CONSTANS (CO)*, a light-sensitive activator of *FT* (Samach et al. 2000, Lazaro et al. 2015). Peak transcript levels of *CO* occur late in the afternoon (Suarez-Lopez et al. 2001). CO is degraded in darkness, which means that in short days, when dusk arrives early, the peak of CO protein accumulation will occur during the dark, and thus the protein degrades. In long days, peak expression coincides with light when CO activates *FT* to induce flowering. *TaHD1* is a wheat *CO* orthologue located on the long arms of chromosome group 6, distal to the *TOC1* locus. Like *CO, TaHD1* exhibits diurnal gene expression (peak during the day, low at night) in long days, suggesting conservation of the day-length-sensing mechanisms between *Arabidopsis* and cereals.

## Light perception

Phytochromes perceive light and so contribute to photoperiod responses and regulation of the circadian clock. There are two interchangeable states of phytochrome chromoprotein, Pr and PFr. The inactive form, Pr, absorbs red light, and the active form, PFr, absorbs light from the far-red regions of the visible spectrum. PFr interacts with phytochrome-interacting factors, helix–loop–helix transcription factors that regulate processes in wheat, like growth responses (towards the direction of sunlight or to minimise shading for example), and flowering (Pearce et al. 2016). The proportion of PFr to the total chromoprotein, known as the phytochrome-photostationary state of the plant, affects architecture (for instance height, tillering capacity and leaf mass per unit area), which is important for light interception, photosynthetic capacity and yield (Evers et al. 2006; Barnes and Bugbee 1991; Casal 1993; Ugarte et al. 2010). Halliday and Davis (2016) suggest that *Arabidopsis* phytochromes are responsive to temperature and play a role in regulating plant temperature response. In wheat, PHYTOCHROME C (PHYC) is the primary phytochrome that provides light input into the photoperiod flowering pathway (Chen et al. 2014). Unlike PHYC in *Arabidopsis*, wheat PHYC is stable and does not require other phytochromes for activity (Monte et al. 2003). In long days, *PHYC* upregulates both *PPD1* and *TaHD1*, accelerating flowering via *FT1* (Chen et al. 2014; Pearce et al. 2016).

## Earliness per se

Genes that influence duration of the wheat lifecycle in conditions where vernalisation and photoperiod requirements have been met, are described as “*Earliness per se”* *(EPS)* loci (Snape et al. 2001). An emerging theme is that many cereal *EPS* genes correspond to components of the circadian clock. The *EPS-3A*^*m*^ gene of *Triticum monococcum* is an orthologue of the *Arabidopsis LUX/PCL* gene (Gawronski and Schnurbusch 2012). Another *EPS* locus, *Eps-A*^*m*^*1*, encompasses a deletion of the wheat *ELF3* gene (Zikhali et al. 2016). Ochagavía et al. (2019) reports that allelic differences at *TaELF3* confer differing levels of sensitivity to temperature; earliness was associated with an increased sensitivity to temperature during the late reproductive phase of development in hexaploid wheat. The same study also revealed temperature-dependent suppression of *TaGI* due to *TaELF3*. Both genes have also been associated with phytochrome-mediated light signalling and the circadian clock (Ford et al. 2016).

### Secondary adaptive traits

Adapted wheat contains allelic combinations of the multiple genes affecting phenology to ensure that the lifecycle is appropriate to the growing conditions. Secondary to this, other traits are also important and must be matched to phenology and the environment.

## Winter hardiness

In cold climates where wheat is sown in autumn, cultivars require a degree of “winter hardiness” to survive freezing temperatures during the vegetative phase. Key to this is the ability to acclimate to cold, whereby freezing tolerance is acquired in response to low temperatures. This can occur in conjunction with the vernalisation response.

Cold acclimation is mediated by C-repeat-binding factors (CBF), also known as dehydration-responsive element binding (DREB) proteins. *CBF/DREBs* contain a DNA motif of approximately 60 amino acids that bind specific promoter elements (CRT-DRE boxes) of target genes leading to their activation, for instance, *late embryogenesis abundant (LEA)* (also known as *dehydrins (DHNS)*) and *cold-regulated (COR)* genes. Li and Chen (1997) found higher accumulation of *DHNS* transcripts in winter cereals subject to cold relative to spring types, given the same cold acclimation treatment. Upregulation of a cereal-specific *COR* gene (*wlt10*) has been reported in response to low temperature, with accumulation of transcripts more rapid and sustained in a cold-tolerant winter background (Ohno et al. 2001). Soon after exposure to cold, *inducer of cbf expression 1* *(ICE1)* is upregulated, followed by expression of *COR* genes some hours later. In *Arabidopsis*, freezing tolerance is related to the subsequent production of cryoprotectants, such as sucrose, raffinose and hydrophilic peptides, which protect membranes against dehydration during freezing (Thomashow 2010). In wheat, changes in the leaf content of lipids, sugars, sugar alcohols and amino acids have been associated with cold acclimation and metabolomics suggested as a measurement tool for chilling and frost tolerance (Cheong et al. 2019).

Not only have *CBFs* been identified as key components of the cold acclimation pathway, they have also been found to contribute to allelic variation. At least 15 *CBFs* have been identified in wheat with an important locus, *FR2* comprising a cluster of *CBF* genes close to *VRN1* on group 5 chromosomes. Copy-number variation of *CBF* genes at *FRA2* was attributed to increased winter hardiness and therefore adaptation in European winter wheat (Wu̎rschum et al. 2016), while variation at the *FRB2* locus was associated with frost tolerance, flowering time and improved yield (Pearce et al. 2013; Badawi et al. 2007; Eagles et al. 2016). The wild-type allele of *FRB2* is often present in winter wheat, and is considered advantageous for adaptation and yield in frost-prone environments, opposed to a large deleted segment frequently found in spring types, which should be beneficial in areas with low-frost risk (Eagles et al. 2016, 2018). Genetic linkage of the *VRN1* and *FR2* loci, and the association of vernalisation sensitivity with particular alleles of *FRB2*, suggests that co-selection of these independent loci is important for adaptation.

There are broader functions for *CBF* genes, including regulation of growth and development. These transcription factors are members of the *APETALA 2/ethylene-responsive element binding* gene family also involved in floral organ identity and drought and salinity stress response (Yamaguchi-Shinozaki and Shinozaki 2006). A controlled condition experiment involving transgenic barley overexpressing *TaDREB2* and *TaDREB3*, showed that plants that constitutively expressed the transgenes grew more slowly, flowered 2–3 weeks later and had changed activity of other *CBFs* and improved frost tolerance (Morran et al. 2011).

The cold acclimation pathway also potentially plays a role in regulating plant architecture. A feature of many winter-type wheats with a high degree of winter hardiness is early prostrate growth habit, where plants have large tiller angles at the vegetative stage of development (Li and Chen 1997). Prostrate plant types in the vegetative stage might confer adaptation to cold and frosty winters by allowing the plant to be covered by a blanket of snow that protects the crop against freezing temperatures.

## Tillering

Aside from prostrate growth habit, another feature of winter wheat is a high degree of tillering, due to the increased duration of the vegetative phase (a vernalisation-requiring wheat will take longer to switch to reproductive development relative to a vernalisation-insensitive plant). A larger number of tillers can increase yield in a high-input (water, nutrient) system due to production of additional fertile spikes. In water-limiting environments however, a higher tiller number may not contribute to increased yield, with additional tillers unable to support fertile spikes. A tiller-reducing gene in wheat, *TIN*, has been identified and studied for yield effects in water-limiting environments of Australia (Richards 1988). To date, there are conflicting reports of the benefit or disadvantage of reduced tillering due to *TIN* in Australian farming systems, and it is likely that the limited number of backgrounds in which the gene has been studied, along with a strong genotype × environment interaction is confounding (Mitchell et al. 2012; Hendriks et al. 2016; Fletcher et al. 2019). Exploring the optimal tillering potential in different phenological types would be interesting.

## Plant height

Final plant height is another developmental trait that influences adaptation. In high-input irrigated farming systems, cultivars with short stature are required to prevent lodging (Sanchez-Garcia and Bentley 2019), whereas taller cultivars are often suited to low-input dryland systems such as the Australian wheat belt (Mathews et al. 2006). A major determinant of the final plant height is the endogenous supply and sensitivity to the hormone gibberellic acid (GA), which is involved in most aspects of development, including germination, vegetative growth, stem elongation and production of flowers and seeds (see Yamaguchi 2008). GA is also implicated in stress response pathways, for example, drought and salinity (Llanes et al. 2016). Other research shows that GA is an important component of the flowering pathway of grasses (MacMillan et al. 2005) and in barley, early flowering triggered by mutations in *HvELF3* requires elevated GA biosynthesis (Boden et al. 2014).

GA promotes growth by an interaction with, and removal of the effect of growth-inhibitory DELLA proteins. In this process, bioactive GA binds to a receptor protein GA-insensitive dwarf 1 (GID1) and DELLA to form a complex that is targeted by an E3 ubiquitin ligase, degrading DELLA (see Sun (2010) for review).

*REDUCED HEIGHT 1* (*Rht-B1*, *RHT1,* chromosome 4BS) and *REDUCED HEIGHT 2* (*Rht-D1, RHT2,* chromosome 4DS) are homoeologous copies of the same DELLA-encoding gene on the B and D genomes. Mutations in these genes give rise to alleles conferring semi-dwarf habit (reduced stem elongation). These mutations create premature stop codons with subsequent truncated proteins unable to form the GA–GID–DELLA complex. Instead of being degraded, DELLA then accumulates and represses growth. Dwarf alleles *Rht-B1b* and *Rht-D1b* have been deployed in plant breeding to develop wheat adapted to environments with high-yield potential. Otherwise known as green revolution genes, they facilitate use of irrigation and nitrogen fertiliser to boost biomass production, harvest index and yield, by ensuring that crops are adapted to high-input farming systems and do not lodge (Peng et al. 1999). Dwarf alleles can be traced to a Japanese landrace, which was introgressed with US germplasm to create the cultivar Norin-10. This germplasm was then deployed by Norman Borlaug in the International Maize and Wheat Improvement Centre (CIMMYT) breeding program. Alleles from Norin-10 then spread to breeding programs throughout the world via cultivars Pitic 62, Penjamo 62 and their progeny. The success of these cultivars is due to their reduced height and also likely improvement in productive tiller number to increase yield (Evans 1975). Other dwarf alleles of *Rht-B1* and *Rht-D1* have been identified at these loci conferring differing levels of height reduction that may be useful for adaptation in different environments (Pearce et al. 2011).

An international trial found that in high-yielding environments, on average, there is no yield penalty associated with *Rht-B1b* and *Rht-D1b* relative to wild-type alleles in near-isogenic tall lines (Mathews et al. 2006). In low-yielding sites however, semi-dwarfs yielded less than the tall wild-type NIL, and so breeding for taller semi-dwarfs, or “short-talls” would be ideal for adaptation and yield in these environments. This result may reflect the disadvantage of dwarf alleles of *Rht-B1* and *Rht-D1* loci, that the whole plant is insensitive to GA. This means that after germination, the growing sheath that delivers the shoot from the seed to soil surface (coleoptile) is also reduced in length. For this reason, *Rht-B1b*- and *Rht-D1b*-carrying lines cannot be sown as deep as their wild-type counterparts. This can have a negative impact on establishment and the ability to capture soil moisture deep in the profile (Whan 1976).

Other dwarfing genes responsive to GA and so with potential to maintain long coleoptiles have been described in wheat (Ellis et al. 2004). Recently, a mutant with lower endogenous GA content (originally described in durum) was identified as *Rht18* on chromosome 6AS (Ford et al. 2018). An agronomic study (Tang 2016) suggests that *Rht18* is a promising candidate to replace *Rht-D1b*. Haque et al. (2011) proposed *that Rht14* and *Rht16* are alleles at the same locus, and based on the map location of *Rht24* in Chinese Spring (Wu̎rschum et al. 2017), it is possible that this gene is allelic to *Rht18*. A distinct locus on chromosome 6A, *Rht25*, reduced height to a lesser extent than *Rht-B1b* and *Rht-D1b* (Mo et al. 2018), and may be a good candidate to produce “short-talls”. Other dwarfing genes, including *Rht4*, *Rht5* and *Rht8*, are attractive breeding targets for adaptation if they are not associated with growth penalties such as short coleoptiles (Ellis et al. 2004).

There is a coincidence of height and phenology, and studies have detected an association of *VRN1* and *PPD1* with plant height in both diverse and structured genetic wheat populations (Camargo et al. 2018; Wilhelm et al. 2013). It is important to consider dwarfing genes and phenological variation together as gene–gene and gene–environment interactions will affect the final plant height.

## Quantitative traits in the farming system

**Fig. 3:**
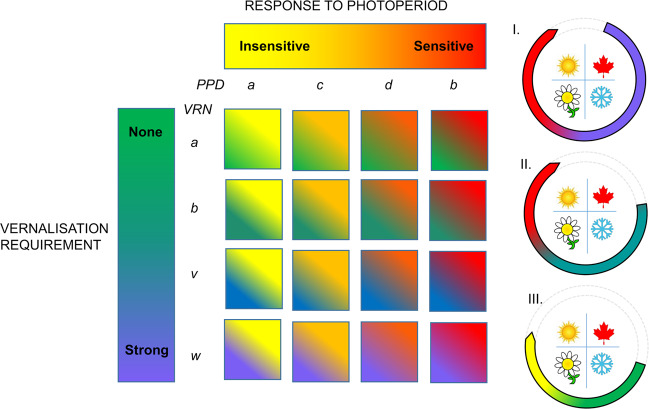
Seasonal lifecycle of wheat: major genes in the farming system. Photoperiod response from insensitive (yellow) to sensitive (red) conferred by alleles (*a, c, d, b*) at *PPD1-D* loci and vernalisation requirement from none (green) to strong (lilac) conferred by alleles (*a, b, v, w*) at *VRN1-A* loci (allele nomenclature from Cane et al. 2013) changes life-cycle duration and adaptation to different growing environments and times of sowing: I. Adaptation to cold winters and early sowing—slow-developing wheat, II. Adaptation to mild winter—mid-developing wheat, III. Adaption to hot summer and late sowing—fast-developing wheat.

Phenology is fundamental to the adaptation of wheat. This is particularly evident in the cropping regions of Australia. Cultivars with a strong vernalisation requirement and sensitivity to day length are suited to regions of Australia which have cold winters and a high risk of frost (Fig. 3). Most of the wheat-growing regions of Australia have milder winters and hot and dry summers, so wheats with a shorter lifecycle from lack of vernalisation requirement and day-length sensitivity (spring types) are traditionally sown after late autumn rain and flower early in spring, before temperature and drought stress in summer (Fig. 3). In response to a changing climate, a field and simulation study assessed performance of different combinations of development alleles in near-isogenic lines (Hunt et al. 2019), and suggested that a shift to earlier sowing of slower-developing genotypes in these regions would increase yield, despite the predicted decrease in rainfall and increase in temperature. For this to occur, Australian breeders need to develop cultivars with slower rates of development and flowering behaviour matched to each growing environment. This is possible with the use of high-throughput marker platforms in breeding programmes to select allelic combinations for adaptation (Grogan et al. 2016). Other traits, such as plant architecture and tolerance to climatic stress, are also important to optimise yield in each farming system. The complex network of genes that underlie adaptation interact strongly with the environment, and in a changing climate, breeding new cultivars and changing agronomic practices will be required to ensure future crop success.

## Future possibilities

The current understanding of phenology and adaptation was developed through reductionist approaches, such as gene mapping in biparental populations, and detailed studies of NILs, to determine the genetic basis and develop molecular markers for individual traits. These approaches are often time consuming and labour intensive. Emerging technologies, including whole-genome sequencing, high-throughput genotyping and genome-wide analytical techniques, are accelerating progress and allow research to be conducted at a larger, more holistic scale. The transcriptome for instance, captures the response of the genome to the environment. Transcriptome analysis of diverse genetic material adapted to different climates around the globe should provide new insights. Other data, such as proteomics and metabolomics, will also be invaluable, and analytical techniques such as machine learning, utilised to handle different types of data at scale. Technologies that allow rapid resolution of complex systems will be important to harness quantitative traits for future crop improvement, particularly where these traits exhibit strong environmental interactions.

## Conclusion

Quantitative traits are complex due to the action of multiple genes and their interactions with each other and the environment, giving rise to a continuous distribution of phenotypes. Phenology and plant architecture are examples of quantitative traits that are fundamental contributors to the adaptation of wheat. Major loci include *VRN, PPD, EPS, RHT* and genes from the *CBF/DREB* family, though there are many other minor-affect loci that are important for adaptation. It is a worthy pursuit to characterise the genes that underlie these traits, and most relevant if the effect of alleles can be assessed in the growing environment that best reflects the farmer’s field. In this way, breeders can target allelic combinations for specific wheat-growing regions and farm management systems. As the global climate changes, new allelic combinations may be required for the adaptation of wheat. For breeders to deliver future adapted cultivars, expedited methods of research to understand gene pathways in relevant environments alongside development of markers for selection are required.
